# Emotion and Implicit Timing: The Arousal Effect

**DOI:** 10.3389/fpsyg.2017.00176

**Published:** 2017-02-14

**Authors:** Sylvie Droit-Volet, Mickaël Berthon

**Affiliations:** Laboratoire de Psychologie Sociale et Cognitive, UMR 6024, CNRS, Université Clermont AuvergneClermont-Ferrand, France

**Keywords:** timing, time, emotion, implicit timing

## Abstract

This study tested the effects of emotion on implicit time judgment. The participants did not receive any overt temporal instructions. They were simply trained to respond as quickly as possible after a response signal, which was separated from a warning signal by a reference temporal interval. In the testing phase, the inter-signal interval was shorter, equal or longer than the reference interval and was filled by emotional pictures (EP) of different arousal levels: high, moderate, and low. The results showed a U-shaped curve of reaction time plotted against the interval duration, indicating an implicit processing of time. However, this RT-curve was shifted toward the left, with a significantly lower peak time for the high-arousal than for the low-arousal EP. This emotional time distortion in an implicit timing task suggests an automatic effect of emotion on the internal clock rate.

## Introduction

In everyday life, we feel that time expands or contracts according to our emotions, for example our feelings of anger or joy toward our loved ones. This fluctuation of time judgment with emotion has been experimentally examined in numerous studies with different emotional stimuli (e.g., affective pictures, facial expressions, sounds). The results have systematically shown time distortions with emotional stimuli, at least when they are highly arousing. Time distortions under the effect of emotions are thus considered as a robust phenomenon (for a review, see Droit-Volet et al., 2013; Lake, 2016). However, there is a debate about the mechanisms underpinning temporal emotional effects. Some authors hold that attentional processes are involved, while others believe they are due to arousal, which automatically increases the rate of the internal clock system.

Most studies on time and emotion in human adults have used explicit time judgment tasks, in which participants are aware that they must estimate time and exercise cognitive control over the contents of their minds. Cognitive executive functions (attention, decision) therefore contribute to time judgment in this type of task. To further examine the idea of an automatic effect of emotion on the internal clock, Droit-Volet (2016) recently decided to use an implicit timing paradigm in which the processing of time is considered to be automatic or, at the least, places only limited demands on attention (Zelaznik et al., 2002; Coull and Nobre, 2008; Merchant et al., 2008; Johnson et al., 2015). In an implicit timing paradigm, the participants do not receive any temporal instruction. For example, they may be presented with two successive signals and then have to press as quickly as possible after the second signal. In this way, it has been demonstrated that the reaction time (RT) for the second signal depends on the length of the interval between the warning and the response signal (foreperiod). The RT is thus faster after a long than a short foreperiod (hazard function: increasing probability of an event with elapsing time) (Niemi and Näätänen, 1981). After a constant inter-signal interval, the RT is also faster for foreperiods close to this reference interval than for shorter or longer foreperiods (Piras and Coull, 2011; Droit-Volet and Coull, 2016). This results in a U-shaped RT curve when the RT is plotted against the different tested foreperiods. The variation of RT as a function of the length of the foreperiod reveals that the motor preparation depends on the temporal interval between the warning and the response signal, and consequently that the duration of this inter-signal interval has been processed. In this type of implicit timing task, we can therefore assume that the U-shaped RT curve will be shifted toward the left under the effect of arousing emotional stimuli if these do indeed automatically increase the rate of the internal clock. According to the internal clock models (for a review, see Wearden, 2016), when the rate of the internal clock increases, more ticks (pulses, oscillations) are emitted per time unit. In a temporal production task, the temporal target is then reached faster, thus resulting in shorter estimates.

To further study this idea, Droit-Volet (2016) thus decided to use an implicit time judgment task and to fill the interval between the two signals with emotional stimuli: i.e., emotional facial expressions (angry, sad and neutral facial expressions). However, the results were not convincing. The RT curves were shifted toward the left and indicated a lower peak time (minimum value of the curve) for the angry than for the sad faces, which were judged to be less arousing. However, no significant difference was observed between the angry and the neutral faces. In addition, and more problematically, the results did not reveal any significant interaction between the emotional stimuli and the foreperiod duration. The RT was always faster in response to angry faces. This is consistent with an automatic acceleration of motor executive functioning in response to an aggressive person, regardless of the inter-signal interval (Woods et al., 2015). In an implicit timing task based on RTs with very short interval durations (<1 s), it is likely that this automatic acceleration of RT for action readiness in a social interaction context (in front aggressive facial expressions) masks the effects of emotion on the processing of durations *per se*. Indeed, the emotional effects on behavior depend on emotional stimuli used and their meaning for individuals (Coan and Allen, 2007). Therefore, to verify the effect of emotion on the RT in an implicit timing task, an implicit timing paradigm similar to that used by Droit-Volet (2016) was used in the present study, but with emotional pictures (EP) from the International Affective Pictures System (IAPS, Lang et al., 1999) which is standardized on the basis of ratings of pleasure and arousal.

The aim of the present study was therefore to test the effect of emotional stimuli on time judgment in an implicit timing task with a constant interval between the warning and the response signal in an initial phase and different inter-signal intervals (equal, shorter, and longer) in a subsequent testing phase (see Piras and Coull, 2011). In this task, the participants always had to press as quickly as possible after the second signal. In our experiment, color EP from the IAPS were selected based on their level of arousal: high, moderate and low. In addition, each participant rated the level of arousal induced by these pictures on the Self-Assessment Manikin scale (SAM, Lang et al., 1999). According to the hypothesis that the internal clock accelerates automatically in the presence of High-Arousal (HA) emotions, we expected to obtain an interaction effect between the inter-signal interval duration and the emotional stimuli on the RT, indicating a leftward shift of the U-shaped RT curve, with a lower peak time, that increases with the increase in the arousal level induced by the EP.

## Materials and Methods

### Participants

Fifty undergraduate psychology students from Clermont Auvergne University (mean age = 25.72, *SD* = 7.52, 36 females and 14 males) participated in this experiment, which was conducted in accordance with the Helsinki declaration. They signed written informed consent before participating in this study, which was approved by the Sud-Est VI statutory ethics committee (CPP, France).

### Material

In a quiet room in our laboratory, the participants were seated in front a computer that controlled the experimental events via E-prime. They responded on the “0” key of the computer’s numeric keypad with the index finger of the dominant hand. A 50-ms auditory stimulus (signal) was given at the onset and the offset of the interval to be timed. Two types of pictures were presented during this interval: a controlled non-emotional picture (no-EP) and an emotional picture. The no-EP was a scrambled black-and-white picture. The emotional pictures (EP) were selected from the IAPS (Lang et al., 1999) for their level of arousal: High-Arousal (HA), Moderate-Arousal (MA) and Low-Arousal (LA). The IAPS pictures for the High-Arousal pictures were a (1) snake (IAPS picture number, n° 1120), (2) a nasty Pitbull (n° 1300), and (3) a shark (n° 1930), for the MA pictures, (1) a sad woman (n° 2271), an elderly woman (n° 2590), and a woman in the rain (n° 9210), and for the LA pictures, (1) a basket (n° 7010), a dustpan, (n° 7040) and an iron (n° 7030). In addition, after the timing task, each participant assessed the arousal level of the different EP they had seen using the 9-point scale of the Self-Assessment Manikin (SAM) (Lang et al., 1999). The pictures were presented on the computer screen until the participants completed the paper SAM scale.

### Procedure

In the timing task, the participants did not receive any overt temporal instruction. They were simply asked to press as quickly as possible after the second auditory signal, which followed the first one and was separated from it by a temporal interval. These two signals were delivered 200 ms after the participant initiated the trial by pressing on the spacebar of the computer keyboard after the word “ready/prêt” presented on the center of the computer screen. The inter-trial interval was randomly chosen between 500 and 1000 ms. The timing task consisted of a training and a testing phase. In the training phase (25 trials), the inter-signal interval was always 500 ms (reference interval duration). The participants were told that they had to learn to press quickly after the second signal. In the testing phase (27 trials), they were only told that they had to continue doing what they had done before. However, the inter-signal interval (probe interval durations) was similar to the reference duration (500 ms), shorter (200, 300, 400 ms) or longer (600, 700, 800 ms) than the reference duration. The probe interval duration similar to the reference duration was presented 9 times (9 trials) and the other probe interval durations were presented 3 times each (3 trials × 6). The trial presentation order was randomized within each trial block (3 blocks of 9 trials).

The participants were assigned to the High-Arousal group (25 participants) or the Moderate-Arousal group (25 participants). In each group, all participants were given two successive sessions with the same timing tasks (training phase and testing phase), except for the EP presented in the second session. In the first session, the picture presented during the inter-signal interval was always the control picture (no-EP) for both the training and the testing phase. In the second session, the control picture was always used for the training phase (no-EP), while EP were presented for the testing phase. In the High-Arousal group, the pictures were the HA and the LA EP. In the MA group, they were the MA and the LA EP. As there were two types of EP during the testing phase (HA vs. LA in the High-Arousal group, and MA vs. LA in the MA group), this led to a total of 54 trials (2 EP × 27 trials). The EP were randomly presented within each block (3 blocks of 9 trials × 2).

## Results

### Emotional Assessment

For each group taken separately, an ANOVA was performed on the mean rating of arousal level for each type of emotional picture. This ANOVA confirmed the effect of emotion for the HA group, *F*(1,24) = 154.50, *p* = 0.0001, $\eta_{p}^{2}$ = 0.87, and the MA group, *F*(1,24) = 34.58, *p* = 0.0001, $\eta_{p}^{2}$ = 0.59. This indicated that the participants in the HA group judged the High-Arousal EP (*M* = 5.80, *SE* = 0.37) to be more arousing than the LA EP (*M* = 1.38, *SE* = 0.23). The participants in the MA group also judged the MA EP (*M* = 4.69, *SE* = 0.37) to be more arousing than the LA EP (*M* = 1.88, ES = 0.23), *F*(1,24) = 154.50, *p* = 0.0001, $\eta_{p}^{2}$ = 0.59. The ANOVA on the arousal ratings for the LA EP with the group as factor found no difference between groups, *F*(1,48) = 2.40, *p* = 0.13, while that on the pictures with a higher arousal level showed an effect of group indicating that the High-Arousal EP were judged more arousing than the MA EP, *F*(1,48) = 4.47, *p* = 0.04, $\eta_{p}^{2}$ = 0.09.

### Reaction Time

**FIGURE 1:**
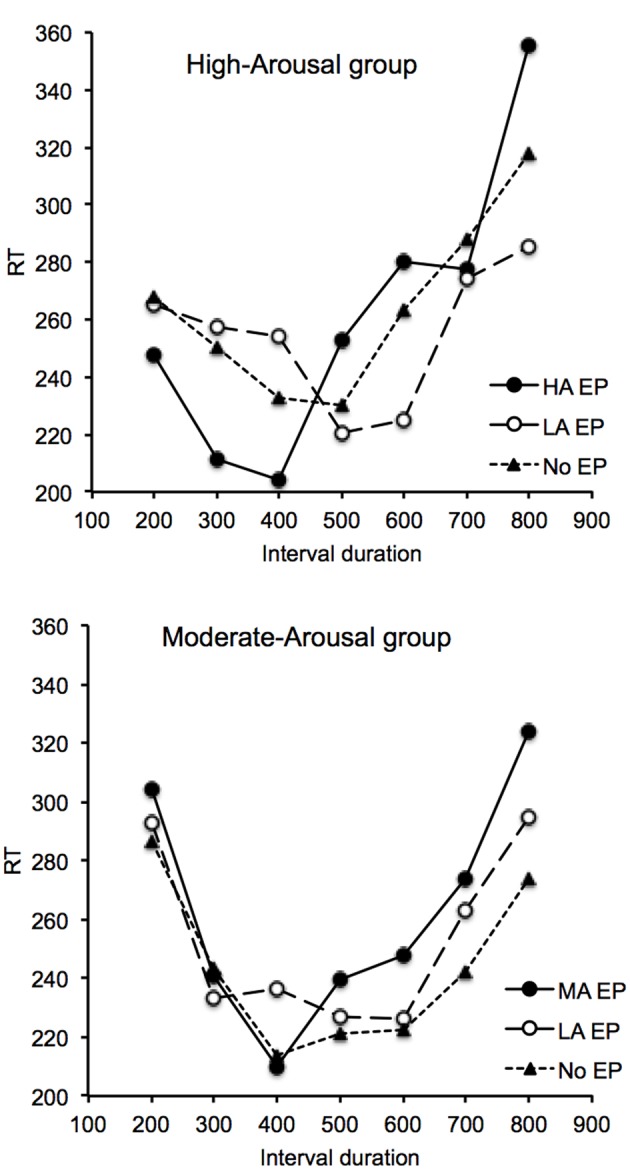
**Mean reaction time (RT) plotted against the probe interval durations for the High-Arousal group with the High-Arousal (HA-EP), the Low-Arousal (LA-EP) emotional pictures and the no-emotional pictures (No-EP), and for the Moderate-Arousal group with the Moderate-Arousal (MA-EP), the Low-Arousal (LA-EP) emotional pictures and the no-emotional pictures (No-EP)**.

The mean RT^1^ was measured on the different trials for each emotional condition. **Figure 1** shows the mean RT plotted against the interval durations in the High-Arousal group (top) and the Moderate-Arousal group (bottom). In all conditions, the RT curves were U-shaped, with RT decreasing to a minimum before increasing again. This demonstrates the persistence of implicit processing of time in different emotional contexts. For the MA group, the ANOVA performed on the RT with two within-subjects factors (emotion and interval duration) found indeed a significant effect of interval duration, *F*(6,144) = 9.26, *p* = 0.0001, $\eta_{p}^{2}$ = 0.28, with a significant quadratic effect, *F*(1,24) = 20.55, *p* = 0.0001, $\eta_{p}^{2}$ = 0.46. The effect of emotion, *F*(2,48) = 1.03, *p* = 0.36, and the interaction between emotion and interval duration were not significant, *F*(12,288) = 0.62, *p* = 0.82. Consequently, in this group, the type of picture presented during the inter-signal interval did not affect the U-shaped RT curve. For the High-Arousal group, the same ANOVA performed on the RT also found a significant main effect of interval duration, *F*(6,144) = 7.71, *p* = 0.0001, $\eta_{p}^{2}$ = 0.24, with a quadratic effect, *F*(1,24) = 21.49, *p* = 0.0001, $\eta_{p}^{2}$ = 0.47. However, in this case, the emotion x interval duration interaction reached significance, *F*(12,288) = 1.77, *p* = 0.05, $\eta_{p}^{2}$ = 0.07, and subsumed no main effect of emotion, *F*(2,48) = 0.19, *p* = 0.83. For the HA group, *post hoc* analyses of the effects on the ANOVA was thus conducted with the interval duration and the emotion (HA-EP vs. LA-EP; no-EP vs. HA-EP; no-EP vs. LA-EP) as factors in order to compare each emotional condition. The ANOVAs systematically showed a main effect of interval duration (all *p* < 0.05), and no main effect of emotion (*p* < 0.05). More interestingly, the emotion x interval duration interaction was significant when the HA EP were compared with the LA EP, *F*(6,144) = 2.95, *p* = 0.01, $\eta_{p}^{2}$ = 0.11. This interaction was not significant for the other comparisons: no-EP vs. LA-EP, *F*(6,144) = 0.65, *p* = 0.69, no-EP vs. HA-EP, *F*(6,144) = 1.46, *p* = 0.20. In sum, there was a significant leftward shift of the U-shaped curve for the HA EP in comparison to the LA EP, with the no-EP curve located halfway between them.

### Peak Time

**FIGURE 2:**
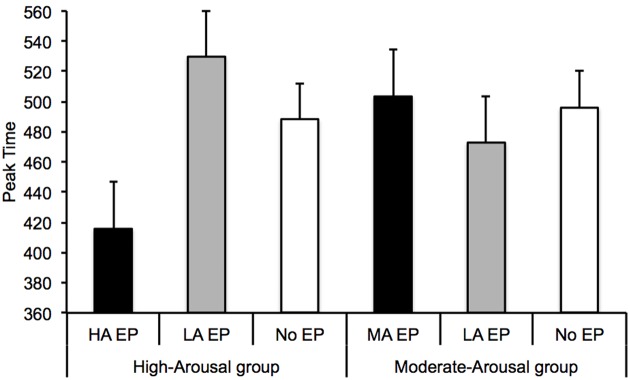
**Peak time of the U-shaped RT curve for the High-Arousal and the Moderate-Arousal group with the High-Arousal/Moderate-Arousal emotional pictures, the Low-Arousal emotional pictures, and the no-emotional pictures**.

To further examine the leftward shift of the RT curves, we measured the peak time (minimum value) of the U-shaped curves by fitting each individual curve with the polynomial function from the GraphPad Prism program. The polynomial function provided reasonably good fits of the temporal curves for most of the participants (all *p* < 0.05). However, the fit was not significant for eight participants in at least one emotional condition. In such cases, we simply took the probe duration corresponding to the lowest RT value. This was not possible for two participants who produced low RT at different probe durations. They were therefore excluded from subsequent statistical analyses. **Figure 2** shows the peak time obtained in this way in the HA and MA groups. The ANOVA performed on the peak time showed a significant effect of emotion for the HA group, *F*(2,46) = 5.25, *p* = 0.009, $\eta_{p}^{2}$ = 0.19, but not for the MA group, *F*(2,46) = 0.44, *p* = 0.65. In the HA group, the peak time was thus lower for the HA EP (*M* = 415.68, *SD* = 157.81) than for the LA EP (*M* = 529.75, *SD* = 159.91), *t*(23) = 3.55, *p* = 0.002, *d* = 0.72. No other significant difference was observed.

In addition, we analyzed the correlation between (1) the difference in the peak time for the LA-EP and that for the more highly arousing pictures (MA or HA) and (2) the difference in the self-arousal rating between these two types of pictures. A significant correlation was observed (*r* = 0.39, *p* = 0.03), demonstrating that the difference in the peak time between the LA and the MA/HA EP increased with the increase in the difference in the arousal assessment of the EP. In other words, the decrease in the peak time of the RT curve for the arousing pictures compared to the LA pictures became greater as the level of experienced arousal increased.

## Discussion

This study investigated the effects of emotion on implicit timing by using EP from the IAPS with different arousal levels. The results showed that the RT was faster for the interval duration close to the reference interval duration in all conditions, thus yielding a U-shaped curve. This finding confirms that the processing of time is not disrupted by the emotional context in an implicit timing task as has been shown in tasks examining explicit time judgment. Human beings therefore maintain their ability to discriminate durations in most contexts.

However, some time distortions occurred with the emotional stimuli. Indeed, our results showed that the U-shaped RT curve was shifted toward the left, with a significantly lower peak time, for the high-arousal emotion compared to the LA emotion. This leftward shift of the RT curve was not observed between emotions of lower arousal levels: i.e., moderate and low. When the individual arousal level was judged low on the 9-point scale of the SAM (<5), no difference in time judgment was observed between the MA, the LA and the control picture (scrambled black-and-white picture). This indicates that significant differences in the perception of time occur only when the difference in the arousal level is sufficiently high. The level of arousal induced by the perception of emotional stimuli is thus one of the major affective dimensions that produce distortions in the perception of durations. Our results showed that the magnitude of the time distortion (leftward shifting of peak time) was indeed positively correlated with the self-reported assessment of arousal level: The more alert the subjects felt in response to arousing pictures (HA, MA), the shorter their estimates were. Our results therefore support the evidence indicating that the effects of emotion on time perception are mediated by arousal (Mella et al., 2010; Dirnberger et al., 2012; Gil and Droit-Volet, 2012; Fayolle et al., 2015). They also support the findings showing that the extent of time distortions depends on individual levels of alertness: i.e., individual emotionality (Tipples, 2011), individual level of anxiety (Bar-Haim et al., 2010; Jusyte et al., 2015; Yoo and Lee, 2015), individual alerting efficiency (Liu et al., 2015), or individual emotional reactivity in response to specific images (spider phobia) (Buetti and Lleras, 2012; Tipples, 2015). However, our study tested the effect of arousal and not that of valence as only stimuli of negative valence were used. The interaction between these two affective dimensions must be examined in future experiments.

In addition, our statistical analyses revealed a significant interaction between emotion and the duration of the interval between the warning and the response signal in the HA group, but without any main effect of emotion. The participants did not therefore always press faster for the highly arousing emotional stimuli, regardless of the inter-signal interval duration. In other words, the effects of emotion on RT varied as a function of the inter-signal interval durations. Consequently, we can conclude that the emotional stimuli directly affected the processing of time in the present study. This significant interaction was not found in the experiment conducted by Droit-Volet (2016) with emotional facial expressions and a similar temporal task (although the auditory signal was 50 ms in our study instead of 100 ms in her study). In Droit-Volet’s experiment, the RT was always faster for the angry faces than for the other faces regardless of the foreperiod. There was thus an automatic increase in executive motor function in preparation to act in response to an aggressive face (Woods et al., 2015). Finally, this specific face-related issue contributed greatly to preventing the emergence of a clear emotional effect on timing in a RT-based task. This highlights the importance for the investigation of temporal emotional effects of the type of emotional stimuli used, of their meaning for individuals, and of the task used (Gil and Droit-Volet, 2012).

The significant interaction between emotion and inter-signal interval duration validates the hypothesis of an automatic acceleration of the internal clock system under the effect of arousal induced by the emotional stimuli. Indeed, arousal enabled the emotional stimuli to speed up the rate of the internal clock, with the result that the time criterion was reached earlier (shorter estimates). Numerous authors defend this clock-based hypothesis (Lake, 2016). Indeed, it is well established that high-arousal situations (threatening situations) produce a release of dopamine in the brain, and that the amount of cerebral dopamine affects the internal clock rate (Cheng et al., 2016). However, our study with an implicit timing task provides additional data suggesting that this emotional clock-related effect is automatic, i.e., it occurs even when the processing of time does not require attention. In other words, the effect of high-arousal emotions on implicit timing would not be mediated by changes in the amount of attention allocated to temporal information. This is in line with the studies showing the persistence of emotional effects on time judgment in children with different attentional abilities (Gil et al., 2007), in tasks of different degrees of difficulty (Droit-Volet et al., 2016) or with masked emotional pictures (Yamada and Kawabe, 2011). However, it is necessary to remember that the automatic or non-automatic nature of the processing of time in implicit temporal tasks, such as the one used in our study, is a subject of discussion (Vallesi et al., 2014). Some authors therefore simply prefer to consider that temporal processing is less attentionally demanding in these tasks (Coull and Nobre, 2008). Consequently, our results suggest that the acceleration of the internal clock under the effect of high-arousal emotion produces time distortions whatever the quantity of attentional resources needed to process time. Obviously this does not mean that attentional processes do not contribute to time judgment in emotional situations. It has been shown that attentional processes (attention detection) trigger emotional reactions to stimuli (Vuilleumier, 2005). Droit-Volet et al. (2015) have also shown that the self-control activity allows the participants to regulate (decrease) the impact of emotion on their time judgments. In conclusion, the effect of high-arousal emotions on time perception due to a speeding-up of the internal clock induced by the increase of arousal was confirmed for implicit timing in the present study just as it has been confirmed for explicit timing in other studies.

## Author Contributions

SD-V conceived and, with MB, designed the experiment. She also analyzed the data and wrote the paper.

## Conflict of Interest Statement

The authors declare that the research was conducted in the absence of any commercial or financial relationships that could be construed as a potential conflict of interest.

## Footnotes

1 The mean percentage of RTs excluded from the analyses (i.e., <50 ms or >1500 ms) was 1.59 (*SD* = 2.53) (see Piras and Coull, 2011).
